# Distinct disruptions of resting-state functional brain networks in familial and sporadic schizophrenia

**DOI:** 10.1038/srep23577

**Published:** 2016-04-01

**Authors:** Jiajia Zhu, Chuanjun Zhuo, Feng Liu, Wen Qin, Lixue Xu, Chunshui Yu

**Affiliations:** 1 Department of Radiology and Tianjin Key Laboratory of Functional Imaging, Tianjin Medical University General Hospital, Tianjin 300052, China; 2 Department of Psychiatry Functional Neuroimaging Laboratory, Tianjin Mental Health Center, Tianjin Anding Hospital, Tianjin 300070, China

## Abstract

Clinical and brain structural differences have been reported between patients with familial and sporadic schizophrenia; however, little is known about the brain functional differences between the two subtypes of schizophrenia. Twenty-six patients with familial schizophrenia (PFS), 26 patients with sporadic schizophrenia (PSS) and 26 healthy controls (HC) underwent a resting-state functional magnetic resonance imaging. The whole-brain functional network was constructed and analyzed using graph theoretical approaches. Topological properties (including global, nodal and edge measures) were compared among the three groups. We found that PFS, PSS and HC exhibited common small-world architecture of the functional brain networks. However, at a global level, only PFS showed significantly lower normalized clustering coefficient, small-worldness, and local efficiency, indicating a randomization shift of their brain networks. At a regional level, PFS and PSS disrupted different neural circuits, consisting of abnormal nodes (increased or decreased nodal centrality) and edges (decreased functional connectivity strength), which were widely distributed throughout the entire brain. Furthermore, some of these altered network measures were significantly correlated with severity of psychotic symptoms. These results suggest that familial and sporadic schizophrenia had segregated disruptions in the topological organization of the intrinsic functional brain network, which may be due to different etiological contributions.

Schizophrenia is an etiologically heterogeneous psychiatric disease, and it has been widely accepted that genetic and environmental factors act in variable proportions in the etiology of schizophrenia^1^. Classification of schizophrenia based on family history may facilitate etiological research of the illness^2^. Schizophrenia patients with a positive family history are classified as ‘familial’ and are considered more likely to have the genetic causes, while those with a negative family history are classified as ‘sporadic’ and are considered more likely to have the environmental causes^3^,^4^. Differences in psychotic symptoms^5^,^6^,^7^,^8^, neurological assessments^9^, neuropsychological tests^10^, psychobiological measures^11^, prevalence of minor physical anomalies^12^, incidence of obstetric complications^13^, age of illness onset^14^,^15^,^16^, season of birth^8^, and outcome^15^ have been reported between patients with familial and sporadic schizophrenia.

Previous neuroimaging studies have mainly focused on structural differences between familial and sporadic schizophrenia. For examples, Schwarzkopf *et al.* reported that familial patients exhibited reduced cranial and cerebral areas, whereas sporadic patients showed marked lateral ventricular enlargement^17^. Roy *et al.* found increased volume of the lenticular nuclei and greater ventricular asymmetry in familial cases compared with sporadic cases and healthy controls^18^. DeQuardo *et al.* found that patients with sporadic schizophrenia had significantly larger ventricle-brain ratio than patients with familial schizophrenia^19^. A voxel-based morphometry (VBM) study has found that only familial patients showed lower gray matter density than controls in the thalamus, suggesting that familial schizophrenia is associated with more severe structural abnormalities than sporadic schizophrenia^20^. A diffusion tensor imaging (DTI) study has revealed that white matter integrity abnormalities in the temporal lobe and corpus callosum were more severe in the sporadic schizophrenia than in the familial schizophrenia^21^. However, little is known regarding the functional differences between familial and sporadic schizophrenia. Only one prior study has shown resting-state cerebral blood flow (CBF) differences between the two subtypes of patients^22^.

Graph theory provides a powerful theoretical framework for characterizing topological properties of brain networks^23^,^24^,^25^. Using these approaches, the complex human brain networks have been found to have a ‘small-world’ topology^26^, which is characterized by a high local specialization and a high global integration, in support of a high efficiency at a low wiring cost^27^,^28^,^29^,^30^,^31^,^32^. Schizophrenia is hypothesized to result from the topological disruptions of such an optimal small-world network (for reviews, see^33^,^34^,^35^,^36^,^37^). The construction and analysis of structural and functional brain networks in schizophrenia have been performed using multiple neuroimaging techniques, including the electroencephalogram (EEG), magnetoencephalogram (MEG), functional magnetic resonance imaging (fMRI), structural MRI and DTI^35^. A wealth of evidence indicates that the brain networks in schizophrenia exhibit altered small-world property, network efficiency, and nodal centrality^33^,^34^,^35^,^36^,^37^, as well as disrupted edges (connectivity)^38^,^39^,^40^. Therefore, a systematical investigation on differences in the topological organization of the brain functional networks between familial and sporadic schizophrenia will facilitate a more sophisticated understanding of the neuropathological mechanisms of the two subtypes of schizophrenia.

On the basis of great differences in clinical symptoms^5^,^6^,^7^,^8^ and brain properties^17^,^18^,^19^,^20^,^21^,^22^ between the two subtypes of schizophrenia, we hypothesize that familial and sporadic schizophrenia would show segregated disruptions in the topological organization of intrinsic functional brain networks. To test our hypothesis, resting-state fMRI data were collected from twenty-six patients with familial schizophrenia, 26 patients with sporadic schizophrenia and 26 healthy controls. We applied graph theoretical approaches to construct and systematically analyze the intrinsic functional brain networks. Inter-group differences in the topological properties of the networks and their relationships with psychotic symptoms were investigated.

## Material and Method

### Participants

A total of seventy-eight right-handed individuals were recruited for this study, including 26 patients with familial schizophrenia (PFS), 26 patients with sporadic schizophrenia (PSS) and 26 healthy controls (HC). The study was conducted in accordance with the Declaration of Helsinki and was approved by the Medical Research Ethics Committee of Tianjin Medical University General Hospital. Written informed consent was obtained from all participants. Diagnosis of schizophrenia was determined by consensus of two experienced psychiatrists using the Structured Clinical Interview for the DSM-IV Axis I Disorder, Patient Edition (SCID-P). All healthy controls were screened using the non-patient edition of the SCID (SCID-NP) to confirm an absence of psychiatric illnesses. The exclusion criteria for all participants were MRI contraindications, the presence of a systemic medical illness (e.g., cardiovascular disease, diabetes mellitus) or central nervous system disorder (e.g., epilepsy) that would affect the study results, a history of head trauma (e.g., hemorrhage), or substance (e.g., hypnotics, alcohol) abuse within the past 3 months or a lifetime history of substance dependence. Additional exclusion criteria for the HC included a history of psychiatric disease and first-degree relatives with a history of psychotic episodes. The Positive and Negative Symptom Scale (PANSS)^41^ was used to assess the severity of psychotic symptoms.

### Family History

Family history was assessed by researchers who were blind to patient information using the Family Interview for Genetic Studies^42^. Information about all first- and second-degree family members was obtained through semi-structured interviews with at least one family informant (e.g., parent, sibling or offspring). Only the family members with schizophrenia-related chronic psychoses (i.e., schizophrenia, schizoaffective, and psychosis not otherwise specified) that were diagnosed with DSM-IV were considered because these diagnoses are more reliable than spectrum personality diagnoses in family history researches. Generally, the PFS was defined as having at least one first- or second-degree family member who had such a schizophrenia-related chronic psychosis, whereas the PSS were those who had no first- or second-degree family members with psychosis. To reduce the risk of misclassification, we employed a stricter definition of PFS where only schizophrenia patients with at least one first-degree family member who had a schizophrenia-related chronic psychosis were classified as PFS.

### Data Acquisition

MRI data were acquired using a 3.0-Tesla MR system (Discovery MR750, General Electric, Milwaukee, WI, USA). Tight but comfortable foam padding was used to minimize head motion, and earplugs were used to reduce scanner noise. Sagittal 3D T1-weighted images were acquired using a brain volume (BRAVO) sequence with the following parameters: repetition time (TR) = 8.2 ms; echo time (TE) = 3.2 ms; inversion time (TI) = 450 ms; flip angle (FA) = 12°; field of view (FOV) = 256 mm × 256 mm; matrix = 256 × 256; slice thickness = 1 mm, no gap; 188 sagittal slices; and acquisition time = 250 s. Resting-state functional blood-oxygen-level-dependent (BOLD) images were acquired using a gradient-echo single-short echo planar imaging (GRE-SS-EPI) sequence with the following parameters: TR/TE = 2000/45 ms; FOV = 220 mm × 220 mm; matrix = 64 × 64; FA = 90°; slice thickness = 4 mm; gap = 0.5 mm; 32 interleaved transverse slices; 180 volumes; and acquisition time = 370 s. All subjects were instructed to keep their eyes closed, relax, move as little as possible, think of nothing in particular, and not fall asleep during the scans. All MR images were visually inspected to ensure that only images without visible artifacts were included in subsequent analyses.

### Data Preprocessing

Resting-state BOLD data were preprocessed using SPM8 (http://www.fil.ion.ucl.ac.uk/spm). The first 10 volumes for each participant were discarded to allow the signal to reach equilibrium and the participants to adapt to the scanning noise. The remaining volumes were corrected for the acquisition time delay between slices. Then, realignment was performed to correct the motion between time points. All participants’ BOLD data were within the defined motion thresholds (i.e., translational or rotational motion parameters less than 2 mm or 2°). We also calculated frame-wise displacement (FD), which indexes the volume-to-volume changes in head position. Several nuisance covariates (six motion parameters, their first time derivations, the global brain signal, the white matter signal, and the cerebrospinal fluid signal) were regressed out from the data. Recent studies have reported that the signal spike caused by head motion significantly contaminated the final resting-state fMRI results even after regressing out the linear motion parameters^43^. Therefore, we further regressed out spike volumes when the FD of the specific volume exceeded 0.5. The datasets were then band-pass filtered in a frequency range of 0.01 to 0.08 Hz. In the normalization step, individual structural images were linearly co-registered with the mean functional image; then the transformed structural images were segmented into gray matter, white matter, and cerebrospinal fluid. The gray matter maps were non-linearly transformed to the tissue probability maps in the Montreal Neurological Institute (MNI) space. Finally, each filtered functional volume was spatially normalized to MNI space using the parameters estimated during the linear co-registration and resampled into a 3–mm cubic voxel.

### Network Construction

GRETNA software (http://www.nitrc.org/projects/gretna) was used to construct the whole-brain functional network. A functional brain network consists of nodes (brain regions) and edges (functional connectivity) between nodes. To define the network nodes, automated anatomical labeling (AAL) template^44^ was employed to segment the whole brain into 90 (45 for each hemisphere) cortical and subcortical regions of interest (ROIs), which were considered a set of nodes in our network analysis. For each subject, the representative mean time series of each ROI was obtained by averaging the BOLD time series over all voxels within that region. To define the network edges, we computed the Pearson correlation coefficients between the regional mean time series of all possible pairs of nodes, resulting in a 90 × 90 correlation matrix for each subject^27^,^32^,^45^. Finally, each correlation matrix was thresholded (see below for the threshold selection) and converted into a binary matrix (i.e., adjacency matrix), where the entry *a*_*ij*_ = 1 if the absolute value of the Pearson correlation coefficient between regions *i* and *j* was larger than the threshold and *a*_*ij*_ = 0 otherwise.

### Network Analysis

We applied a range of sparsity thresholds, which was defined as the ratio of the number of existing edges divided by the maximum possible number of edges in a network, to all correlation matrices. This approach guaranteed that all resultant networks would be comprised of the same number of edges, thereby enabling us to test the inter-group differences in relative network organization^46^,^47^. In our study, a sparsity threshold range of 0.10 to 0.34 with an interval of 0.01 was employed according to several previous studies^48^,^49^,^50^. This thresholding strategy was determined such that the generated networks were estimable for the small-worldness and had sparse properties with the minimum possible number of spurious edges^48^,^49^,^50^.

For brain networks at each sparsity threshold, both global and regional network measures were calculated. The global measures included five small-world property metrics^26^ and two network efficiency metrics^46^,^51^. The small-world property metrics^26^ consisted of clustering coefficient *C*_*p*_ (a measure of the extent of the local density or cliquishness of the network), characteristic path length *L*_*p*_ (a measure of the extent of average connectivity or overall routing efficiency of the network), normalized clustering coefficient *γ* (the ratio of the clustering coefficients between real and random networks), normalized characteristic path length *λ* (the ratio of the characteristic path length between real and random networks), and small-worldness *σ* = *γ*/*λ* (scalar quantitative measurement of the small-worldness of a network). The network efficiency metrics^46^,^51^ were comprised of global efficiency *E*_*glob*_ (a measure of the global efficiency of parallel information transfer in the network) and local efficiency *E*_*loc*_ (a measure of the fault tolerance of the network). The regional measures included the degree, betweenness, and efficiency (see a review^52^ for uses and interpretations). We calculated the area under the curve (AUC) for each network metric, which provided a summarized scalar for the topological characterization of brain networks. Since the integrated AUC metric is independent of a single threshold selection and is sensitive to topological alterations of brain disorders, it has been extensively used in brain network studies^47^,^48^,^49^,^50^,^53^.

To further examine the edge metric (i.e., functional connectivity strength) of the network, we used the network-based statistics (NBS) method (http://www.nitro.org/projects/nbs/)^39^. Firstly, we identified the nodes that exhibited significant inter-group differences in at least one of the three nodal metrics (the degree, efficiency, and betweenness). Then, a functional connectivity matrix was created for each subject on the basis of these altered nodes. Subsequently, the NBS method was utilized to localize those neural circuits showing significant changes in functional connectivity strength.

### Statistical Analysis

To determine whether there were significant differences in the topological properties of the functional brain networks across the PFS, PSS and HC, a one-way analysis of variance (ANOVA) was performed on the AUC of each network metric (small-world property, network efficiency and nodal metrics). Because these analyses were exploratory in nature, we used a statistical significance level of *P *< 0.05. In the NBS analysis, we applied nonparametric permutation tests to identify significant inter-group differences in functional connectivity strength (threshold = 2.0, *P *< 0.05, 10000 permutations). Because global signal regression (GSR) is a controversial topic in resting-state functional MRI analyses^54^,^55^, we also repeated our analysis using fMRI data without GSR.

Once significant inter-group differences were identified in any network metrics, we further assessed the relationships between these metrics and PANSS scores in the PFS and PSS, respectively. A partial correlation analysis was used to test the association with age and sex as the nuisance covariates. The statistical significance level was set at *P *< 0.05.

## Results

### Demographic and Clinical Characteristics of Subjects

The demographic and clinical characteristics of the sample are shown in Table 1. The PFS, PSS and HC were well-matched in age (one-way ANOVA, *F*_2,75_ = 0.016, *P* = 0.984) and sex (chi-square test, Pearson’s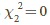, *P* = 1). There were no significant differences between PFS and PSS in terms of antipsychotic dosage (two sample t-test, *t*_50_ = 0.808, *P* = 0.423), duration of illness (two sample t-test, *t*_50_ = 0.887, *P* = 0.379) and PANSS scores (two sample t-test; PANSS positive score, *t*_50_ = −1.318, *P* = 0.194; PANSS negative score, *t*_50_ = −1.639, *P* = 0.107; PANSS general score, *t*_50_ = −1.881, *P* = 0.066; PANSS total score, *t*_50_ = −1.974, *P* = 0.054). None of the schizophrenia patients had experienced electroconvulsive therapy.

### Intergroup Differences in Global Measures

**Figure 1:**
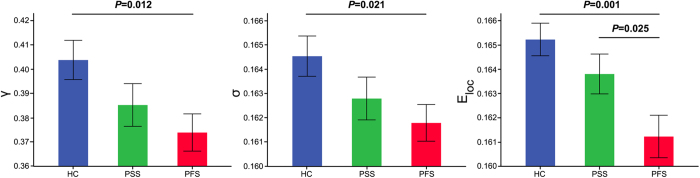
The differences in global measures of the networks across the PFS, PSS and HC. Error bars represent standard errors. *E*_*loc*_, local efficiency; HC, healthy controls; PFS, patients with familial schizophrenia; PSS, patients with sporadic schizophrenia; *γ*, normalized clustering coefficients; *σ*, small-worldness.

In the defined threshold range, functional brain networks of the PFS, PSS and HC exhibited higher clustering coefficients (i.e., *γ* > 1) but almost identical characteristic path lengths (i.e., λ ≈ 1) relative to comparable random networks, which indicates that the three groups showed a typical small-world topology (i.e., *σ* > 1) (Fig. S1). Although all the three groups satisfied small-world topology, one-way ANOVA revealed significant inter-group differences in both small-world property and network efficiency (Fig. 1). Compared with the HC, the PFS showed significantly lower normalized clustering coefficient *γ* (*P *= 0.012) and small-worldness *σ* (*P *= 0.021). No significant differences were observed in *γ* and *σ* between the PSS and the HC (*γ*, *P *= 0.114; *σ*, *P *= 0.139), and between the PFS and the PSS (*γ*, *P *= 0.331; *σ*, *P *= 0.392). In addition, there were no significant inter-group differences in clustering coefficient *C*_*p*_, characteristic path length *L*_*p*_, or normalized characteristic path length *λ*. With regard to network efficiency, the PFS exhibited significantly decreased local efficiency *E*_*loc*_ compared to both the PSS (*P *= 0.025) and HC (*P *= 0.001). However, there was no difference in *E*_*loc*_ (*P *= 0.210) between the PSS and HC. Additionally, no significant difference in global efficiency *E*_*glob*_ was identified across the three groups.

### Intergroup Differences in Regional Measures

**Figure 2:**
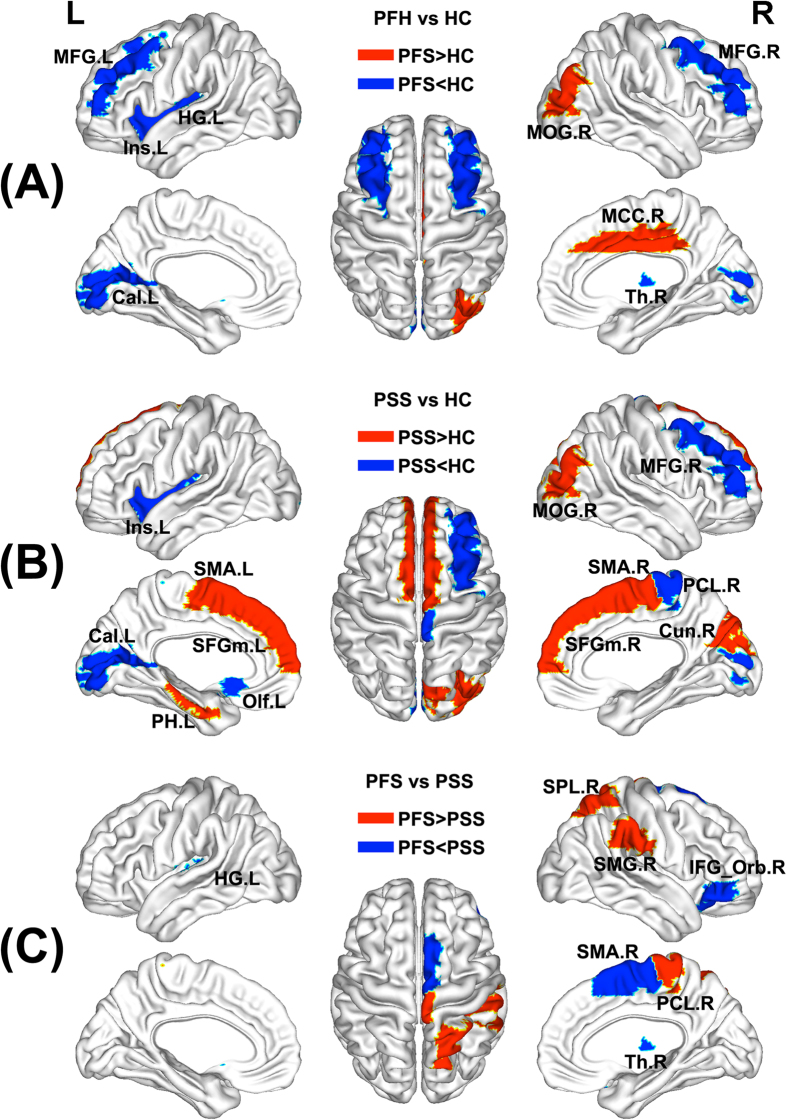
The differences in regional measures of the networks across the PFS, PSS and HC. See Table S1 for the detailed information. The subcortical regions showing group differences in nodal centralities are not shown here. Cal, calcarine gyrus; Cau, caudate; Cun, cuneus; HC, healthy controls; HG, Heschl’s gyrus; IFG_Orb, orbital part of inferior frontal gyrus; Ins, insula; L, left; MCC, mid-cingulate cortex; MFG, middle frontal gyrus; MOG, middle occipital gyrus; Olf, olfactory cortex; PCL, paracentral lobule; PFS, patients with familial schizophrenia; PH, parahippocampal gyrus; PSS, patients with sporadic schizophrenia; Put, putamen; R, right; SFGm, medial part of superior frontal gyrus; SMA, supplementary motor area; SMG, supramarginal gyrus; SPL, superior parietal lobule; Th, thalamus.

Brain regions exhibiting significant inter-group differences in at least one nodal metric were identified (Fig. 2, Table S1). Compared with the HC, the PFS exhibited increased nodal centralities in the right mid-cingulate cortex (MCC) and middle occipital gyrus (MOG), and decreased nodal centralities in the bilateral middle frontal gyrus (MFG), the left insula (Ins), calcarine gyrus (Cal), caudate (Cau), putamen (Put) and Heschl’s gyrus (HG), and the right thalamus (Th) (Fig. 2A, Table S1). Compared with the HC, the PSS showed increased nodal centralities in the bilateral supplementary motor area (SMA) and medial part of the superior frontal gyrus (SFGm), the left parahippocampal gyrus (PH), and the right cuneus (Cun) and MOG, and decreased nodal centralities in the left olfactory cortex (Olf), Ins and Cal, and the right MFG, paracentral lobule (PCL) and Put (Fig. 2B, Table S1). A direct comparison between the PFS and PSS revealed that the PFS showed increased nodal centralities in the right superior parietal gyrus (SPL), supramarginal gyrus (SMG) and PCL, and decreased nodal centralities in the left Cau and HG, and the right orbital part of inferior frontal gyrus (IFG_Orb), SMA and Th as compared with the PSS (Fig. 2C, Table S1).

### Intergroup Differences in Network Measures without GSR

The results of inter-group comparisons in network measures using fMRI data without GSR are shown in *SI Text* and Fig. S2–4 in the Supplementary Materials.

### Intergroup Differences in Functional Connectivity Strength

**Figure 3:**
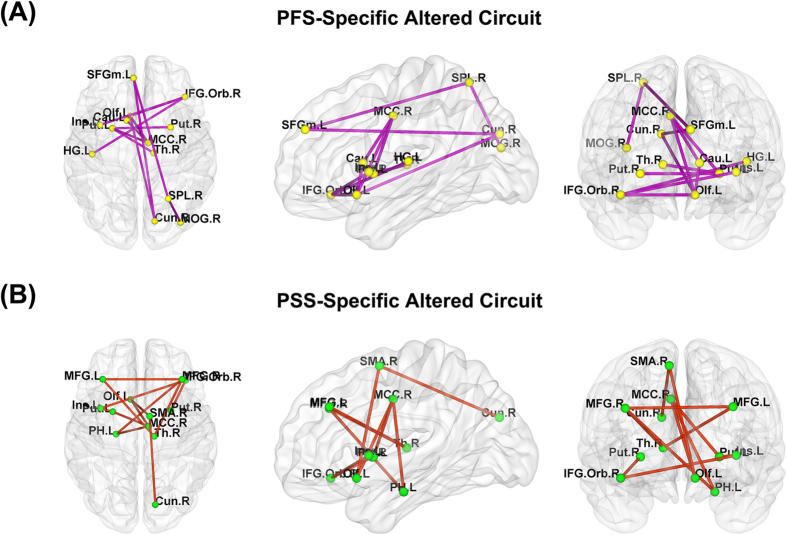
The differences in functional connectivity strength of the networks across the PFS, PSS and HC. The NBS method was used to identify a PFS-specific altered circuit with 13 nodes and 13 edges (*P *= 0.022, corrected) (**A**), and a PSS-specific altered circuit with 12 nodes and 12 edges (*P *= 0.033, corrected) (**B**). The PFS-specific altered circuit consisted of edges exhibiting decreased functional connectivity strength in the PFS compared with the HC; the PSS-specific altered circuit consisted of edges showing decreased functional connectivity strength in the PSS relative to the HC. See Table S2 for the detailed information. Cau, caudate; Cun, cuneus; HC, healthy controls; HG, Heschl’s gyrus; IFG_Orb, orbital part of inferior frontal gyrus; Ins, insula; L, left; MCC, mid-cingulate cortex; MFG, middle frontal gyrus; MOG, middle occipital gyrus; Olf, olfactory cortex; PFS, patients with familial schizophrenia; PH, parahippocampal gyrus; PSS, patients with sporadic schizophrenia; Put, putamen; R, right; SFGm, medial part of superior frontal gyrus; SMA, supplementary motor area; SPL, superior parietal lobule; Th, thalamus.

We utilized the NBS method to identify a PFS-specific altered circuit with 13 nodes and 13 edges (*P *= 0.022, corrected) (Fig. 3A, Table S2), and a PSS-specific altered circuit with 12 nodes and 12 edges (*P *= 0.033, corrected) (Fig. 3B, Table S2). Within the PFS-specific altered circuit, all of the edges exhibited decreased functional connectivity strength in the PFS compared with the HC; within the PSS-specific altered circuit, all of the edges showed decreased functional connectivity strength in the PSS relative the HC. The nodes of the PFS-specific altered circuit included the bilateral Put, the left SFGm, Ins, Olf, Cau and HG, and the right IFG_Orb, MCC, Th, SPL, Cun and MOG (Fig. 3A); the nodes of the PSS-specific altered circuit included the bilateral MFG and Put, the left Ins, Olf and PH, the right IFG_Orb, SMA, MCC, Th and Cun (Fig. 3B). However, a direct comparison between the PFS and PSS revealed that there was no difference in functional connectivity strength between the two patient subgroups.

### Relationships between Network Measures and Psychotic Symptoms

**Figure 4:**
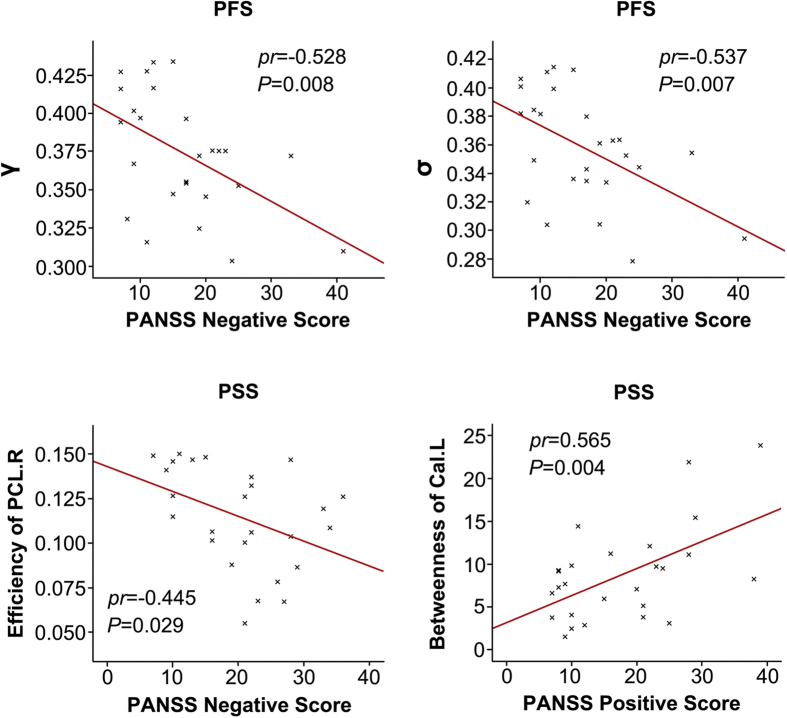
The correlations between the network metrics and the PANSS scores in the PFS and PSS, respectively. Cal, calcarine gyrus; L, left; PANSS, The Positive and Negative Syndrome Scale; PCL, paracentral lobule; PFS, patients with familial schizophrenia; PSS, patients with sporadic schizophrenia; R, right; *γ*, normalized clustering coefficients; *σ*, small-worldness.

The relationships between network metrics and severity of psychotic symptoms are illustrated in Fig. 4. With regard to global metrics, the PANSS negative score was negatively correlated with normalized clustering coefficient *γ* (*pr *= −0.528, *P *= 0.008) and small-worldness *σ* (*pr *= −0.537, *P *= 0.007) in the PFS. As to regional metrics, the PANSS negative score was negatively correlated with the efficiency of the right PCL (*pr *= −0.445, *P *= 0.029) in the PSS; the PANSS positive score was positively correlated with the betweenness of the left Cal (*pr *= 0.565, *P *= 0.004) in the PSS. There were no significant correlations between the PANSS scores and any other global and regional metrics (*P* > 0.05).

## Discussion

The present study is, to our knowledge, the first to apply graph theoretical approaches to compare differences in the topological organization of functional brain networks between patients with familial and sporadic schizophrenia. Three main findings are revealed: (1) at a global level, only the PFS showed significantly lower values in normalized clustering coefficient, small-worldness, and local efficiency, implying a randomization shift of their brain networks; (2) at a regional level, PFS and PSS disrupted different neural circuits, suggesting distinct neural mechanisms between the two subgroups; and (3) these altered network measures were significantly correlated with severity of psychotic symptoms, indicating potential biomarkers of the subtypes of the disorder.

The human brain is characterized by an economical small-world network with high local clustering and short path length, corresponding to an intermediate state between regular and random networks^27^,^28^,^29^,^30^,^31^,^32^. In contrast, the regular network has high local clustering and long path length, whereas the random network has low local clustering and short path length^26^. Compared with healthy controls, patients with familial schizophrenia exhibited reduced clustering efficient and local efficiency but unchanged characteristic path length and global efficiency, suggesting that the network configuration shifts towards a random network organization. This randomization shift of the brain networks in schizophrenia has been consistently reported in several previous studies^56^,^57^,^58^,^59^,^60^,^61^. Either reduced local connections or increased long-distance connections may lead to network randomization. Our finding of reduced clustering efficient and local efficiency but unchanged characteristic path length and global efficiency supports the former. More importantly, our findings provide evidence that the familial schizophrenia, rather than the sporadic schizophrenia, exhibit the schizophrenia-related network randomization. Moreover, the randomization shift of the brain networks is clinically relevant, that is, the reduced clustering efficient was negatively correlated with the PANSS negative score in patients with familial schizophrenia. This finding suggests that a greater network randomization may predict more severe negative symptoms. Thus, the clustering coefficient and small-worldness may be potential biomarkers for monitoring the illness progress in patients with familial schizophrenia.

In addition to the global topologies, we also investigated the node and edge attributes of the functional brain network. The nodal centralities (degree, efficiency and betweenness) reflect the central roles of nodes in the overall information communication of the networks^62^. The edge attribute reflects the functional connectivity strength between a pair of nodes. All the changes in regional properties of the functional network reflect abnormalities in regional neural circuits within the network, which may provide additional information that cannot be derived from the investigation on the global topology of the network. These node and edge properties have been extensively utilized to characterize several brain diseases, such as major depressive disorder^50^ and posttraumatic stress disorder^48^,^49^. We found functional connectivity strength reduction in widespread brain regions involving the default mode network (DMN), visual network (VN), auditory network (AN), salience network (SN), sensorimotor network (SMN), central executive network (CEN), and subcortical network. These regions are extensively involved in introspection and episodic memory (DMN), multimodal sensory processing (VN, AN, and SMN), executive control and working memory function (CEN), identifying salient stimuli and switching between CEN and DMN (SN). The finding of widespread functional disconnectivity in schizophrenia is consistent with previous whole-brain resting-state functional connectivity studies^38^,^39^. For instance, schizophrenia patients have been shown to exhibit extensive functional connectivity reduction in the brain^38^. Using a NBS method, a prior study has revealed an expansive connectivity strength reduction in schizophrenia, including connections among the frontal, occipital and temporal regions^39^. More importantly, we found that the disrupted neural circuits were completely different in familial and sporadic schizophrenia, suggesting that the neural mechanisms underlying familial and sporadic schizophrenia are largely different. The distinct disconnected neural circuits may account for differences in psychotic symptoms^5^,^6^,^7^,^8^, neurological assessments^9^, neuropsychological tests^10^, and psychobiological measures^11^ between patients with familial and sporadic schizophrenia. Moreover, sporadic schizophrenia patients showed a significant negative correlation between nodal efficiency of the right paracentral lobule (reduced in patients) and the PANSS negative score, indicating that a lower nodal efficiency of this region is related to a more severe negative symptom. Although the betweenness of the left calcarine gyrus was decreased in patients, we also found a positive correlation between this nodal property and the PANSS positive score in sporadic schizophrenia patients. These findings suggest that mild abnormality of the calcarine gyrus may lead to the development of the positive symptom, whereas excessive abnormality might preclude the formation of the positive symptom. Alternatively, the seemingly more “normal” nodal centrality of the calcarine gyrus may be the result of exposure to the more severe positive symptoms.

The present study has several limitations. First, we cannot completely eliminate the potential effects of duration of illness, medication and psychotic symptoms on our results, although there were no significant differences in these clinical variables between familial and sporadic schizophrenia. In future researches, medication-naïve, first-episode schizophrenia patients with more homogeneous clinical features are needed to validate the findings of this study. Second, we did not make any genetic tests to confirm the PFS such that we cannot rule out the possibility of heterogeneity in PFS which may affect our results. Third, we calculated the network measures based on a binary adjacency matrix. Future studies may consider performing a weighted network analysis which might provide additional information. Fourth, the functional brain networks were constructed at a coarsely regional level by segmenting the whole brain into 90 regions based on the AAL template. Several studies have pointed out that different parcellation strategies may lead to considerable variations in the graph theoretical metrics^63^,^64^,^65^. Future work should use different parcellation schemes to test the reproducibility of our results. Finally, the nodal centrality analyses were not corrected for multiple comparisons because of the modest sample size. In the future, a larger sample of participants is required to increase statistical power of comparative analysis.

In conclusion, this is the first study to systematically compare differences in the topological properties of the functional brain networks between familial and sporadic schizophrenia using resting-state fMRI and graph theoretical approaches. Compared with healthy controls, only familial schizophrenia patients exhibited abnormalities in the global properties, characterized by a decreased local specialization and a comparable global integration, indicative of a randomization shift of their brain networks. In regard to the regional properties, patients with familial and sporadic schizophrenia disrupted different neural circuits. These findings suggest that different etiology may be related to distinct disruption in the topological organization of the functional brain network in familial and sporadic schizophrenia. The family history of schizophrenia should be considered as an important factor in future studies on neural mechanisms of schizophrenia.

## Additional Information

**How to cite this article**: Zhu, J. *et al.* Distinct disruptions of resting-state functional brain networks in familial and sporadic schizophrenia. *Sci. Rep.* **6**, 23577; doi: 10.1038/srep23577 (2016).

## Supplementary Material

Supplementary Information

## Figures and Tables

**Table 1:** Demographic and Clinical Characteristics of the Participants.

Characteristics	PFS (n* *= 26)	PSS (n* *= 26)	HC (n* *= 26)	*P*-value
Age (years)	32.5 ± 7.0	32.7 ± 7.0	32.4 ± 7.5	0.984^a^
Sex (female/male)	13/13	13/13	13/13	1^b^
Antipsychotic dosage (mg/d) (chlorpromazine equivalents)	436.4 ± 349.6	373.2 ± 191.0	NA	0.423^c^
Duration of illness (months)	137.3 ± 95.6	114.7 ± 88.1	NA	0.379^c^
PANSS				
Total	60.6 ± 18.3	72.0 ± 23.2	NA	0.054^c^
Positive score	14.8 ± 5.4	17.6 ± 9.6	NA	0.194^c^
Negative score	16.6 ± 8.3	20.4 ± 8.3	NA	0.107^c^
General score	29.2 ± 7.9	34.0 ± 10.4	NA	0.066^c^

The data are shown as the mean ± SD. HC, healthy controls; NA, not applicable; PANSS, The Positive and Negative Syndrome Scale; PFS, patients with familial schizophrenia; PSS, patients with sporadic schizophrenia.

^a^One-way ANOVA was used to test the difference in age across the three groups.

^b^Chi-square test was used to test the difference in sex across the three groups.

^c^Two-sample t-test was used to compare the differences in antipsychotic dosage, duration of illness and PANSS scores between the PFS and PSS.
