# Supplementary material for: Distinct disruptions of resting-state functional brain networks in familial and sporadic schizophrenia
Source: Sci Rep. 2016 Apr 1;6:23577. doi: 10.1038/srep23577 (PMC4817042; doi:10.1038/srep23577)
Supplement: Supplementary Information [file srep23577-s1.doc]

**Full Title:**

**Distinct disruptions of resting-state functional brain networks in familial and sporadic schizophrenia**

**Jiajia Zhu ^1, 3^, Chuanjun Zhuo ^1, 2, 3^, Feng Liu ^1^, Wen Qin ^1^, Lixue Xu ^1^, and Chunshui Yu ^1^, ***

**Running Title:**

**Disrupted functional brain networks in familial and sporadic schizophrenia**

^1^ Department of Radiology and Tianjin Key Laboratory of Functional Imaging, Tianjin Medical University General Hospital, Tianjin 300052, China

^2^ Department of Psychiatry Functional Neuroimaging Laboratory, Tianjin Mental Health Center, Tianjin Anding Hospital, Tianjin 300070, China

^3^ These authors contributed equally to the article.

***Correspondence to:**

Chunshui Yu. Prof. M.D.

Department of Radiology, Tianjin Medical University General Hospital, No. 154, Anshan Road, Heping District, Tianjin 300052, China.

Phone: +86-22-63062026

Fax: +86-22-63062290

E-mail: [chunshuiyu@tijmu.edu.cn](mailto:chunshuiyu@tijmu.edu.cn)

**Supplementary Materials**

**SI Text**

Without GSR, the functional networks of all the three groups still satisfied small-world topology (i.e., γ>1, λ≈1 andσ>1) (Fig. S2). However, the between-group differences in both global and regional measures without GSR were largely different from those with GSR (Fig. S3 and S4), indicating that GSR significantly affects our results.

The global signal has been thought to reflect non-neuronal noise (e.g., physiological, movement, scanner-related) and GSR has been used as a standard step during the processing of resting-state fMRI data^1^. Recently, GSR has been considered a controversial topic in resting-state functional MRI analyses^2,3^ because global signal has also been found to reflect neurobiologically important information^4^. This is confirmed by a previous study reporting increased global signal variability in schizophrenia but not in bipolar disorder^5^. Our findings are consistent with a recent study reporting that GSR can greatly affect between-group differences in resting-state functional connectivity^6^. Taken together, the effects of GSR on resting-state fMRI analyses in schizophrenia are rather complex and need further investigation.

**References**

1 Macey, P. M., Macey, K. E., Kumar, R. & Harper, R. M. A method for removal of global effects from fMRI time series. *Neuroimage* 22, 360-366, doi:10.1016/j.neuroimage.2003.12.042 (2004).

2 Fox, M. D., Zhang, D., Snyder, A. Z. & Raichle, M. E. The global signal and observed anticorrelated resting state brain networks. *J Neurophysiol* 101, 3270-3283, doi:10.1152/jn.90777.2008 (2009).

3 Murphy, K., Birn, R. M., Handwerker, D. A., Jones, T. B. & Bandettini, P. A. The impact of global signal regression on resting state correlations: are anti-correlated networks introduced? *Neuroimage* 44, 893-905, doi:10.1016/j.neuroimage.2008.09.036 (2009).

4 Scholvinck, M. L., Maier, A., Ye, F. Q., Duyn, J. H. & Leopold, D. A. Neural basis of global resting-state fMRI activity. *Proc Natl Acad Sci U S A* 107, 10238-10243, doi:10.1073/pnas.0913110107 (2010).

5 Yang, G. J. *et al.* Altered global brain signal in schizophrenia. *Proc Natl Acad Sci U S A* 111, 7438-7443, doi:10.1073/pnas.1405289111 (2014).

6 Wang, D. *et al.* Altered functional connectivity of the cingulate subregions in schizophrenia. *Transl Psychiatry* 5, e575, doi:10.1038/tp.2015.69 (2015).

**
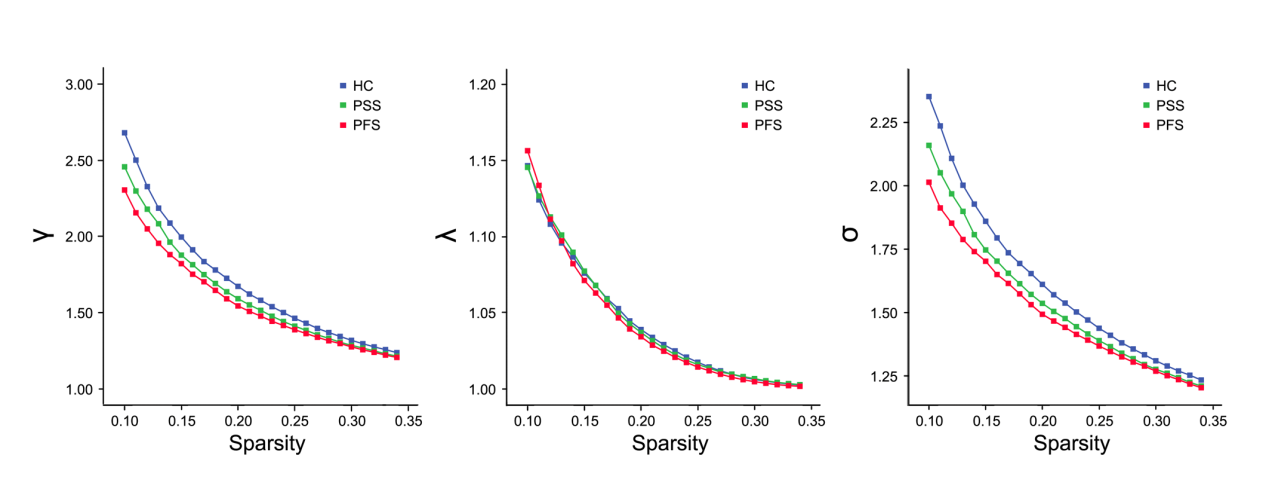
**

**Fig. S1.** The small-world property metrics as a function of sparsity thresholds. In the defined threshold range, the PFS, PSS and HC showed normalized clustering coefficients γ and small-worldness σ substantially greater than 1 and normalized characteristic path lengths λ approximately equal to 1, indicating that the three groups exhibited the typical feature of small-world topology. HC, healthy controls; PFS, patients with familial schizophrenia; PSS, patients with sporadic schizophrenia; γ, normalized clustering coefficients; λ, normalized characteristic path lengths; σ, small-worldness.

**
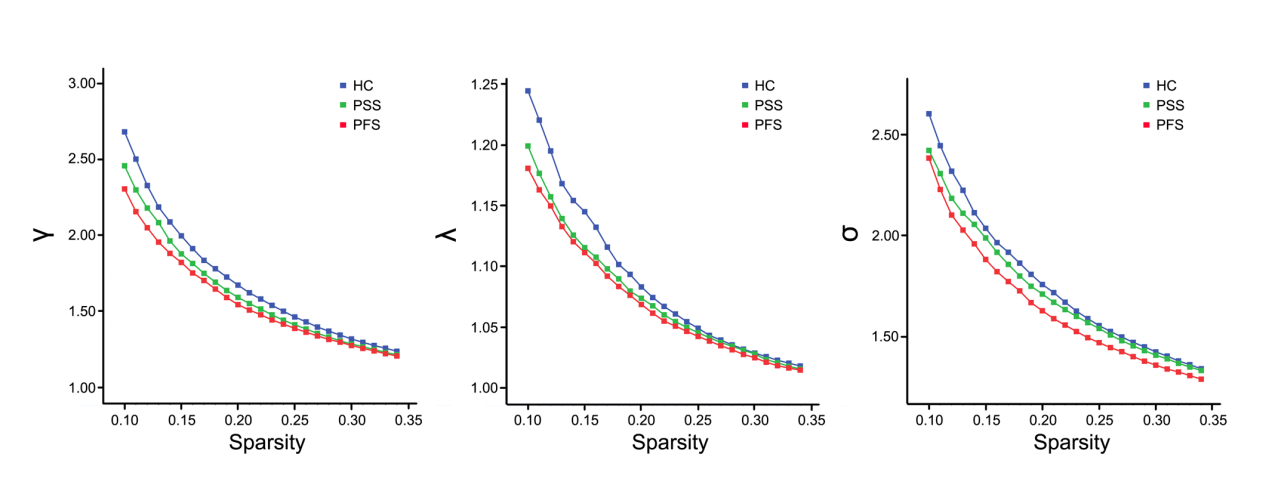
Fig. S2.** The small-world property metrics as a function of sparsity thresholds for functional networks without GSR. In the defined threshold range, the PFS, PSS and HC showed normalized clustering coefficients γ and small-worldness σ substantially greater than 1 and normalized characteristic path lengths λ approximately equal to 1, indicating that the three groups exhibited the typical feature of small-world topology. HC, healthy controls; PFS, patients with familial schizophrenia; PSS, patients with sporadic schizophrenia; γ, normalized clustering coefficients; λ, normalized characteristic path lengths; σ, small-worldness.


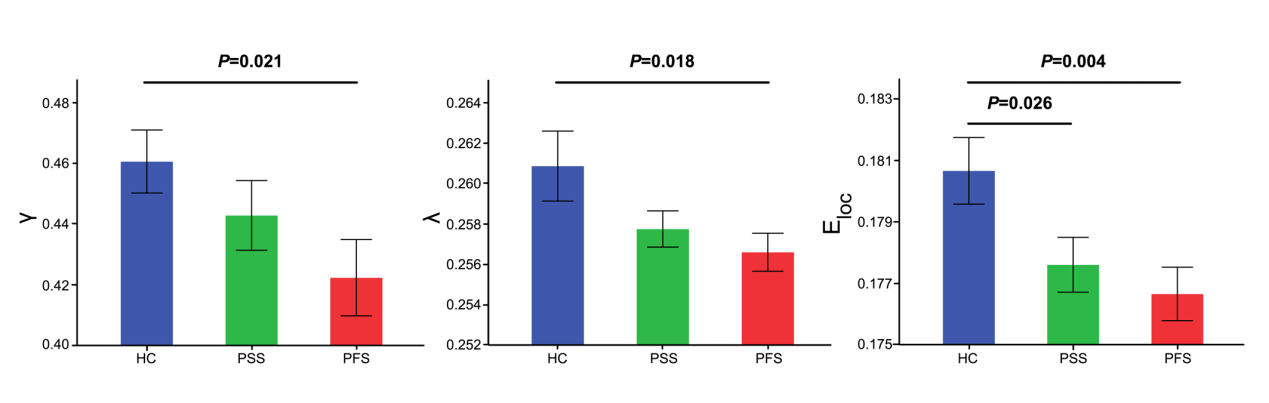
**Fig. S3.** The differences in global measures of the functional networks without GSR across the PFS, PSS and HC. Error bars represent standard errors. Eloc, local efficiency; HC, healthy controls; PFS, patients with familial schizophrenia; PSS, patients with sporadic schizophrenia;γ, normalized clustering coefficients; σ, small-worldness.


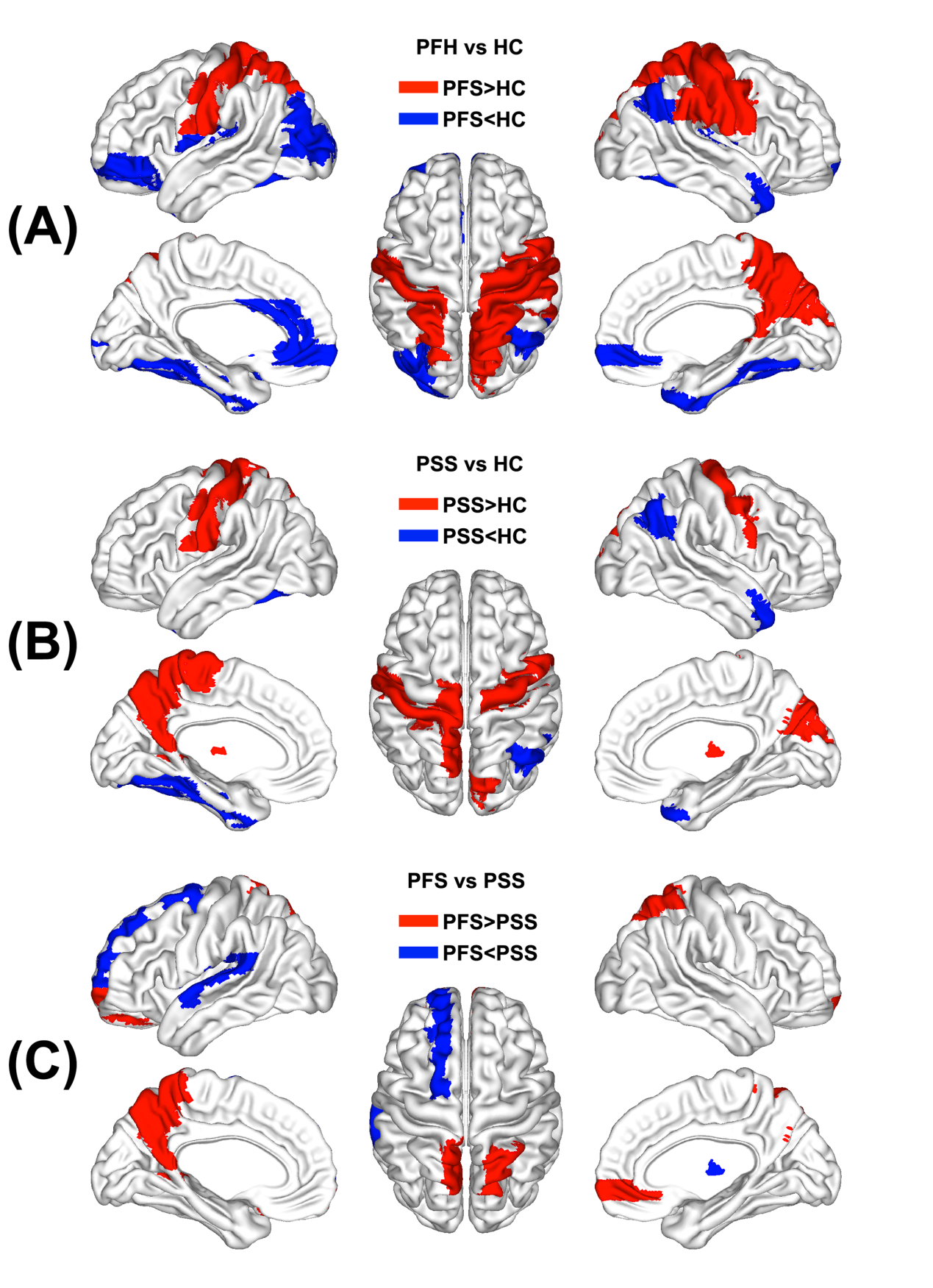


**Fig. S4.** The differences in regional measures of the functional networks without GSR across the PFS, PSS and HC. HC, healthy controls; PFS, patients with familial schizophrenia; PSS, patients with sporadic schizophrenia

**Table S1**. Regions showing significant differences in nodal centralities across the PFS, PSS and HC

|  | *P* Values | | |
| --- | --- | --- | --- |
| Brain Regions | Degree | Betweenness | Efficiency |
| **PFS>HC** | | | |
| MCC.R | 0.163 | **0.046** | 0.186 |
| MOG.R | **0.011** | 0.081 | **0.006** |
| **PFS<HC** | | | |
| MFG.L | **0.042** | 0.128 | **0.047** |
| MFG.R | **0.002** | **0.047** | **0.007** |
| Ins.L | 0.063 | **0.023** | 0.062 |
| Cal.L | 0.881 | **0.032** | 0.894 |
| Cau.L | **0.045** | 0.976 | 0.109 |
| Put.L | 0.125 | **0.041** | 0.260 |
| Th.R | **0.029** | 0.163 | 0.066 |
| HG.L | **0.023** | 0.154 | **0.036** |
| **PSS>HC** | | | |
| SMA.L | 0.115 | **0.024** | 0.066 |
| SMA.R | **0.034** | 0.187 | **0.021** |
| SFGm.L | 0.055 | **0.007** | 0.070 |
| SFGm.R | 0.088 | **0.015** | 0.088 |
| PH.L | **0.028** | 0.197 | 0.051 |
| Cun.R | **0.044** | **0.027** | 0.102 |
| MOG.R | **0.023** | 0.187 | **0.020** |
| **PSS<HC** | | | |
| MFG.R | **0.016** | 0.226 | **0.028** |
| Olf.L | 0.700 | **0.014** | 0.558 |
| Ins.L | 0.227 | **0.018** | 0.184 |
| Cal.L | 0.131 | **<0.001** | 0.093 |
| PCL.R | 0.058 | 0.218 | **0.009** |
| Put.R | 0.089 | **0.007** | 0.121 |
| **PFS>PSS** | | | |
| SPL.R | **0.040** | 0.604 | **0.018** |
| SMG.R | **0.034** | 0.223 | **0.017** |
| PCL.R | 0.125 | 0.119 | **0.021** |
| **PFS<PSS** | | | |
| IFG_Orb.R | 0.057 | **0.023** | 0.102 |
| SMA.R | **0.026** | 0.132 | **0.047** |
| Cau.L | **0.012** | 0.252 | **0.019** |
| Th.R | **0.007** | 0.158 | 0.099 |
| HG.L | **0.027** | 0.191 | **0.034** |

Brain regions exhibiting significant inter-group differences (*P*<0.05) in at least one of the three nodal centralities are listed in the Table. Cal, calcarine gyrus; Cau, caudate; Cun, cuneus; HC, healthy controls; HG, Heschl’s gyrus; IFG_Orb, orbital part of inferior frontal gyrus; Ins, insula; L, left; MCC, mid-cingulate cortex; MFG, middle frontal gyrus; MOG, middle occipital gyrus; Olf, olfactory cortex; PCL, paracentral lobule; PFS, patients with familial schizophrenia; PH, parahippocampal gyrus; PSS, patients with sporadic schizophrenia; Put, putamen; R, right; SFGm, medial part of superior frontal gyrus; SMA, supplementary motor area; SMG, supramarginal gyrus; SPL, superior parietal lobule; Th, thalamus.

**Table S2.** Altered functional connectivity strength across the PFS, PSS and HC

| Functional connections | t-value | Functional connections | t-value |
| --- | --- | --- | --- |
| **PFS-specific altered circuit** | | **PSS-specific altered circuit** | |
| MOG.R-SPL.R | 3.21 | SMA.R-Cun.R | 2.01 |
| SFGm.L-SPL.R | 2.23 | SMA.R-Olf.L | 2.28 |
| SFGm.L-Cun.R | 2.27 | MFG.L-MFG.R | 2.06 |
| MCC.R-Put.L | 2.86 | MFG.L-Th.R | 2.19 |
| MCC.R-Cau.L | 2.13 | MCC.R-Put.L | 3.14 |
| Olf.L-MCC.R | 2.41 | MCC.R-PH.L | 2.10 |
| Olf.L-Cun.R | 3.41 | Olf.L-MCC.R | 2.29 |
| Olf.L-Cau.L | 3.16 | MFG.R-PH.L | 2.31 |
| Put.L-Th.R | 2.28 | MFG.R-Th.R | 2.41 |
| Put.L-Put.R | 2.68 | Ins.L-Put.L | 2.15 |
| IFG_Orb.R-HG.L | 2.80 | IFG_Orb.R-Ins.L | 2.43 |
| IFG_Orb.R-Ins.L | 2.03 | IFG_Orb.R-Put.R | 2.12 |
| IFG_Orb.R-Olf.L | 2.02 |  |  |

The PFS-specific altered circuit consisted of edges exhibiting decreased functional connectivity strength in the PFS compared with the HC; the PSS-specific altered circuit consisted of edges showing decreased functional connectivity strength in the PSS relative to the HC. Cau, caudate; Cun, cuneus; HC, healthy controls; HG, Heschl’s gyrus; IFG_Orb, orbital part of inferior frontal gyrus; Ins, insula; L, left; MCC, mid-cingulate cortex; MFG, middle frontal gyrus; MOG, middle occipital gyrus; Olf, olfactory cortex; PFS, patients with familial schizophrenia; PH, parahippocampal gyrus; PSS, patients with sporadic schizophrenia; Put, putamen; R, right; SFGm, medial part of superior frontal gyrus; SMA, supplementary motor area; SPL, superior parietal lobule; Th, thalamus.
